# Evaluating healthy cities: A scoping review protocol

**DOI:** 10.1371/journal.pone.0276179

**Published:** 2022-10-20

**Authors:** Michelle Amri, Safa Ali, Geneviève Jessiman-Perreault, Kathryn Barrett, Jesse Boardman Bump

**Affiliations:** 1 Takemi Program in International Health, Harvard T.H. Chan School of Public Health, Harvard University, Boston, Massachusetts, United States of America; 2 Dalla Lana School of Public Health, University of Toronto, Toronto, Ontario, Canada; 3 Department of Health and Society, University of Toronto Scarborough, Scarborough, Ontario, Canada; 4 University of Toronto Scarborough Library, Scarborough, Ontario, Canada; 5 Bergen Centre for Ethics and Priority Setting, University of Bergen, Bergen, Norway; University College London, UNITED KINGDOM

## Abstract

**Background:**

The Healthy Cities project supports municipal policymakers in the struggle to safeguard the health of urban citizens around the world (and in other limited geographies such as islands). Although Healthy Cities has been implemented in thousands of settings, no synthesis of implementation experiences have been conducted. In this article, we develop a scoping review protocol that can be applied to collect evidence on process evaluations of Healthy Cities.

**Methods:**

To develop a scoping review protocol that could identify experiences evaluating the Healthy Cities project, we followed the PRISMA guidelines for Scoping Reviews (PRISMA-ScR). We applied these guidelines in consultation with a research librarian to design a search of the peer-reviewed literature, specifically Ovid Medline, Ovid Embase, Web of Science Core Collection, and Scopus databases, and a grey literature search.

**Discussion:**

In addition to the aim of collecting evidence on Healthy Cities process evaluation experiences, the broader goal is to spark discussions and inform future evaluations of Healthy Cities. This work can also inform other evaluations of initiatives seeking to raise socio-political change, such as those focused on enhancing intersectoral and multisectoral action.

## Background

“A Healthy City is one that is continually creating and improving those physical and social environments and expanding those community resources which enable people to mutually support each other in performing all the functions of life and in developing their maximum potential” [1, pp. 4].

The Healthy Cities Movement [2] began in 1986 [3] and spurred the Healthy Cities project, or interchangeably, the Healthy Cities program [4]. The project uses the ‘settings approach’ to health promotion, which entails considering health in the settings of everyday life [5], in other words, where people “learn, work, play, and love” [6]. The goal of the Healthy Cities project is to increase the capacity of municipal governments to address the health of their citizens [7] and in other limited geographies such as islands. Thus, the Healthy Cities project seeks to raise public health on the agenda of urban policymakers [2] and is centered on intersectoral collaboration locally [4] and community action [8]. Key elements of the project include: increasing awareness of urban health issues among municipal and national governments; creating a network of cities to allow for knowledge exchange; and initiating political mobilization and community participation through the development of partnerships across various sectors of municipal government, universities, nongovernmental organizations, community groups, and the private sector [7].

The World Health Organization (WHO) has promoted Healthy Cities across member states. From the initial eleven European cities selected for the project in 1986, this quickly grew to 650 cities in 1993 [2], to over 1400 cities being either covered by a Healthy Cities network or a flagship city (as cited by WHO) [9] and over four thousand participating communities, towns, and cities (as cited by a researcher at the WHO Collaborating Centre for Research on Healthy Cities in 2001) [10]. Although the Healthy Cities project is more established in Europe, there are projects globally, such as in Tehran and Accra, and there are several Healthy Cities networks supported by various WHO Regional Offices [7]. There are thousands of Health Cities projects [3] but thus far there has been no attempt to synthesize implementation experiences.

### Rationale

Given that Healthy Cities entails various theoretical elements, including: “proximal and distal determinants of health, proximal and distal interventions for health, and ‘known impact’”; this complicates evaluation [8, pp. i23]. O’Neill and Simard [3] have suggested that limited recognition of the political nature of evaluation is one factor in explaining why so few evaluations have been done for Healthy Cities projects; the required stakeholder support can be extensive, as with Europe’s Phase V evaluation, which engaged Healthy Cities representatives, the WHO, and academics [11]. For instance, different perspectives on indicators are evident when looking at an early evaluation of the Healthy Cities project in Chittagong, Bangladesh from 1994. This evaluation highlighted the limited utility in impact indicators as substantial time is required to collect these measures, whereas process evaluations can work to quickly ameliorate issues and keep morale high [2]. And further, considered quantitatively, with Europe’s Phase I evaluation having 53 indicators and decreasing to 32 indicators in Phase V—and nevertheless, facing contestation in cities [11]. Evidently, there are various ways an evaluation of Healthy Cities can be undertaken. In fact, it has been noted that creativity is needed to assess the worth of a Healthy Cities Project [12]. In light of these contentions, we present a protocol to synthesize evidence on how process evaluations of Healthy Cities have been conducted and their findings.

### Objective

We are interested understanding approaches taken to evaluate the approach itself across various settings (i.e., process evaluation). We are not interested in evaluating the relationship between the Healthy City projects on individual-level outcomes associated with behaviour change or similar (e.g., tobacco use, alcohol use, unhealthy diet, physical inactivity). In other words, we are interested in assessing impacts (and/or ways of working to achieve such impacts, such as processes or structures) rather than outcomes. An impact is “a result or change that has come about as the result of complex interactions between a range of factors, including Healthy City designation, but potentially much wider (multi-causality, such as socio-political change, economic up/downturns, extending or limiting the range of stakeholders, etc.) and is driven by priorities determined by the Healthy Cities value system” (11); whereas an outcome is “is a result or change that can be directly attributed to such an activity or intervention within a Healthy City […] Or more succinctly: outcome evaluation assesses the result in terms of achievement of objectives set” (11). Because ‘The’ Healthy City does not exist, as each city operates within its own unique context [10], we are not expecting to collate a universal list of indicators [3]. Instead, our aim is to spark discussions and inform future approaches through collating information on how evaluations have been conducted to-date.

In addition to this primary aim, we are also interested in understanding if consideration is afforded to the context in which the city operates, such as cities of the global south, and if so, how this consideration is built into evaluation. This is supported by the aforementioned evaluation from Chittagong, which determined that Healthy Cities projects established in developing countries are fundamentally different than those in industrialized nations, calling for both the need to adapt projects and conduct comparative analysis of Healthy Cities projects in developing countries [2].

While Healthy Cities seems to have an inherent tension between research and practice [13], evaluating Healthy Cities is important for several reasons. These reasons include: assessing any changes to political processes, community health status, or others; sustaining the political legitimacy of Healthy Cities; affording comparison across cities; drawing on successes to maintain community mobilization and participation; and advancing scientific knowledge [3].

## Methods

To design a scoping review protocol that could identify experiences evaluating the Healthy Cities project, we drew on the work of Arksey and O’Malley [14], whereby five primary stages informed the study design: (i) identifying the research question; (ii) identifying relevant studies; (iii) study selection; (iv) charting the data; and (v) collating, summarizing, and reporting the results. This scoping review protocol was developed following the PRISMA guidelines for Scoping Reviews (PRISMA-ScR) [15].

### (i) Identifying the research question

This study protocol can be applied to synthesize evidence on how evaluations of Healthy Cities (including healthy municipalities, healthy communities, healthy islands, and others) have been conducted through drawing on both peer-reviewed and grey literature. Thus, asking: how has the Healthy Cities project or approach been evaluated?

Considering that “the real success of any Healthy City project is when it ceases to be a project, because the system it set up to ensure that health issues are given priority, to involve all stakeholders and to ensure that all sectors recognize that their role in healthy cities becomes part of the structure of local governance” [16, pp. 27], the inclusion and exclusion criteria for this study have been developed to not only better understand how Healthy Cities have been evaluated, but how evaluations also assess such institutionalization structures.

### Inclusion criteria

As such, the inclusion criteria eligibility is as follows: (i) must discuss the healthy cities project/approach (including healthy municipalities, healthy communities, healthy islands, and others), (ii) describe an evaluation undertaken or report results of an evaluation of the approach itself applied within a city, cities, community, etc., and (iii) available in English due to resource constraints.

### Exclusion criteria

References are excluded if they are either (i) not evaluating the Healthy City approach itself, (ii) evaluating health outcome indicators (e.g., physical activity rates) rather than an evaluation of the approach itself—and cases where the Healthy City approach was evaluated using various indicators that may include health outcomes will be included, (iii) evaluating a program that is not a Healthy City project, despite seeking to improve health (i.e., uses language of “healthy city” or “healthy community” without aligning with or referencing “Healthy Cities”), (iv) commentary, reflection, or interview discussing evaluation issues (i.e., not an evaluation with described methods), or (v) the full text is not available.

### (ii) Identifying relevant studies

The search strategy was developed with an academic librarian possessing relevant expertise of appropriate databases and comprehensive search strategies for knowledge synthesis projects. The strategy was designed to cast a wide net, searching a multitude of databases and grey literature from the WHO, namely the: Ovid MEDLINE, Ovid EMBASE, Web of Science Core Collection, Scopus, and WHO Institutional Repository for Information Sharing (IRIS) databases. These numerous databases will be searched to minimize any potential gaps, as the Ovid databases are focused on biomedicine, whereas the Web of Science Core Collection and Scopus draw from a range of disciplines, thus ensuring articles that may be missed through searching biomedical databases will be retrieved in interdisciplinary databases, and vice versa. In addition, through searching the WHO’s IRIS, grey literature hits, specifically WHO texts, will be included that would not be retrieved through these four aforementioned databases. The search strategy will include relevant keywords such as healthy city/healthy cities, healthy municipality/municipalities, healthy community/communities, healthy island(s), evaluation, assessment, effectiveness, efficacy, and indicator(s), and the subject heading of program evaluation. No publication date limit will be applied, but results will be filtered so that only English language results are included. The databases to be searched and associated search strategies are provided in Table 1.

**Table 1:** Databases, rationale, and associated search strings.

Database	Rationale	Search strategy	
Ovid MEDLINE	Ovid MEDLINE will be searched due to the inclusion of biomedicine and life science journals.	**#**	**Search Statement**
		1	((healthy city or healthy cities or healthy municipality or healthy municipalities or healthy community or healthy communities or healthy island or healthy islands) adj10 (program* or project* or initiative* or network* or movement* or world health organi#ation)).tw,kf.
		2	Program Evaluation/
		3	(evaluat* or assess* or effectiveness or efficacy or indicator*).tw,kf.
		4	2 or 3
		5	1 and 4
		6	limit 5 to english language
Ovid EMBASE	Ovid EMBASE has a wide-ranging focus on biomedical evidence, including the areas of health policy and public health, and has a greater number of international journals which is helpful for this review’s global scope.	**#**	**Search Statement**
		1	((healthy city or healthy cities or healthy municipality or healthy municipalities or healthy community or healthy communities or healthy island or healthy islands) adj10 (program* or project* or initiative* or network* or movement* or world health organi#ation)).tw,kw.
		2	exp program evaluation/
		3	(evaluat* or assess* or effectiveness or efficacy or indicator*).tw,kw.
		4	2 or 3
		5	1 and 4
		6	limit 5 to english language
Web of Science	Web of Science will be searched due to its multidisciplinary contents from a range of academic disciplines.	TOPIC: ((("healthy city" OR "healthy cities" OR "healthy municipality" OR "healthy municipalities" OR "healthy community" OR "healthy communities" OR "healthy island" OR "healthy islands") NEAR/10 (program* OR project* OR initiative* OR network* OR movement* OR "world health organi?ation"))) AND TOPIC: ((evaluat* OR assess* OR effectiveness OR efficacy OR indicator*))	
		Refined by: LANGUAGES: (ENGLISH)	
		Timespan: All years. Indexes: SCI-EXPANDED, SSCI, A&HCI, CPCI-S, CPCI-SSH, BKCI-S, BKCI-SSH, ESCI. (Web of Science Core Collection)	
Scopus	Scopus will be searched due to its multidisciplinary nature, which includes the fields of medicine and social sciences.	(TITLE-ABS-KEY ( ( ("healthy city" OR "healthy cities" OR "healthy municipality" OR "healthy municipalities" OR "healthy community" OR "healthy communities" OR "healthy island" OR "healthy islands") W/10 (program* OR project* OR initiative* OR network* OR movement* OR "world health organi?ation") ) ) AND TITLE-ABS-KEY ( (evaluat* OR assess* OR effectiveness OR efficacy OR indicator*) ) ) AND (LIMIT-TO (LANGUAGE, "English") )	
WHO Institutional Repository for Information Sharing (IRIS)	IRIS will be searched for grey literature of WHO texts.	A search using the keyword “healthy cities” will be conducted. The filters menu will be used to refine the search results; “healthy cities” will be entered in the title field and “program evaluation” will be entered in the Subject (MeSH) field. To ensure a comprehensive search, the browse function in IRIS will also be used to review all resources under the subject of “healthy cities.”	

### (iii) Study selection

Covidence software [17] will be used to remove duplicates and undertake the first screen of references by two independent reviewers. This entails two reviewers independently reading the titles and abstracts of all references and screening in any that may be relevant to the research question for full-text review. In the case of the grey literature where abstracts are often unavailable, executive summaries, table of contents, or similar will be screened, following the grey literature protocol utilized by Godin et al. [18]. In the case where these may be unavailable, the grey literature will be advanced to full-text screen. Any conflicts arising from the first screen will be resolved through discussion by the two independent reviewers. In the second stage, the list of references marked for full-text review will be read by two independent reviewers to assess alignment with the inclusion criteria and determine the final references marked for inclusion in the study. Discrepancies will be discussed and resolved by the review team.

Studies marked for inclusion will be further assessed through ‘citation chaining’ (i.e., circling back to stage iii to ensure a comprehensive identification of studies). This entails both reviewing sources cited in the included references and checking resources that cite these included studies to determine if there are any potentially missed references that meet the inclusion criteria. This will be undertaken with Web of Science automatically when this stage is reached.

### (iv) Charting the data

Data extracted from the full texts will be charted across: title, author(s), journal or book title, location of evaluation, method of evaluation (e.g., case study, interviews, focus groups), who conducted the evaluation, indicators assessed/what was evaluated, rationale for the evaluation, and consideration for city context. These dimensions were informed by the work of O’Neill and Simard [3] and de Leeuw [19], and may be adjusted during the review process. For example, additional dimensions deemed worthy of consideration during the review process may be included. Or similarly, dimension(s) that yield minimal information may be excluded.

### (v) Collating, summarizing, and reporting the results

The PRISMA diagram will be used to illustrate the review process and delineate stages where studies were eliminated with the associated reason. The charted data will be narratively synthesized, drawing on the dimensions to thematically group findings in the discussion of the manuscript. We hypothesize being able to point to both commonalities and differences across evaluations and raise important considerations for those undertaking an evaluation of a Healthy Cities project.

## Limitations

Given the resource constraints faced in undertaking this study, as only literature available in English will be assessed, we understand that this is a limitation to this work. Similarly, despite undertaking a systematic approach to reviewing grey literature, given the vast amount of grey literature available, it is possible there may be missed sources. However, in seeking to overcome this difficulty, we intend to publish the findings of the completed review to make the findings more easily accessible for researchers and policymakers.

## Discussion

Given that the Healthy Cities approach has been implemented in thousands of settings but faces the problem of limited synthesized evaluations, we believe it is crucial to gain a better grasp of the landscape in which this inconsistency operates. Our pursuit of assessing the nature of process evaluations in the Healthy Cities projects is supported by other scholars, such as those who have emphasized the importance of evaluation for examining efforts and justifying investments made [3]. Given the political nature of health, better understanding dimensions of policy and governance are crucial [20]. This proposed scoping review of Healthy Cities process evaluations seeks to do this, given that it is a political program [13] (e.g., raising health on the municipal agenda and promoting intersectoral ways of working). Thus, we believe our findings will also be useful for other intersectoral and multisectoral ways of working, such as the Health in All Policies (HiAP) approach [21], including the possible bridging of Healthy Cities with HiAP [22].

## Supporting information

S1 Checklist (DOCX)

## List of abbreviations

HiAP: Health in All Policies
IRIS: Institutional Repository for Information Sharing
PRISMA-ScR: Preferred Reporting Items for Systematic Reviews and Meta-Analyses guidelines for Scoping Reviews
WHO: World Health Organization
